# Expression of CPEB4 in Human Glioma and Its Correlations With Prognosis

**DOI:** 10.1097/MD.0000000000000979

**Published:** 2015-07-13

**Authors:** Wanming Hu, Yuanzhong Yang, Shaoyan Xi, Ke Sai, Dongfang Su, Xinke Zhang, Suxia Lin, Jing Zeng

**Affiliations:** From Department of Pathology, Cancer Center, Sun Yat-Sen University, Guangzhou, China (WH, YY, SX, XZ, SL, JZ); State Key Laboratory of Oncology in South China, Cancer Center, Sun Yat-Sen University, Guangzhou, China (WH, YY, SX, KS, DS, XZ, SL, JZ); Collaborative Innovation Center for Cancer Medicine, Guangdong, China (WH, YY, SX, KS, DS, XZ, SL, JZ); Department of Neurosurgery, Cancer Center, Sun Yat-Sen University, Guangzhou, China (KS); and Department of Clinical Nutrition, Cancer Center, Sun Yat-Sen University, Guangzhou, China (DS).

## Abstract

CPEB4 plays an important role in cancer progression. However, the clinicopathological significance of CPEB4 expression to glioma and its expression levels in glioma tissues and cell lines are unknown. The present study investigated the potential prognostic value of CPEB4 for human glioma.

Immunohistochemistry (IHC) was performed to examine the dynamics of CPEB4 expression in glioma and nonneoplastic brain tissues, and the expression of CPEB4 in cell lines and freshly prepared tissue samples was measured using Western blotting and real-time PCR.

CPEB4 was highly expressed at the mRNA and protein levels in 4 glioma cell lines and in 4 freshly prepared glioma tissues. Immunohistochemical analysis demonstrated that CPEB4 expression in glioma tissue was higher than that in corresponding nonneoplastic brain tissue (*P* < 0.01). This high expression level was further increased in high-grade gliomas, and the CPEB4 expression level correlated with the WHO classification (r = 0.774, *P* < 0.01). Moreover, the overall survival of glioma patients displaying high CPEB4 protein expression (*P* < 0.01) was clearly lower than that of those displaying low CPEB4 expression, and the high CPEB4 expression indicated a poorer survival in high-grade glioma patients (*P* < 0.01).

Our study suggests that CPEB4 is significantly expressed in human glioma and that the upregulation of CPEB4 protein is significantly associated with advanced WHO grade. CPEB4 may serve as a highly sensitive prognostic indicator for glioma patients.

## INTRODUCTION

Glioma is the most common and aggressive type of primary brain tumor, accounting for 30% of all brain and central nervous system tumors and 80% of all malignant brain tumors.^1^ Numerous tumor grading systems are used, but the most common system used for glioma is the World Health Organization (WHO) classification, which grades glioma from I to IV; this grade is further stratified into well-differentiated low-grade glioma (WHO grade I–II) and high-grade glioma (anaplastic glioma, WHO grade III, and glioblastoma, WHO grade IV).^2^ The overall prognosis for glioma is closely related to the WHO grade. The prognosis of malignant glioma remains extremely poor despite recent significant advances in treatment using a combination of multidisciplinary therapeutic approaches, such as surgery and chemotherapy with or without radiotherapy. Indeed, the median survival duration is less than 1 year,^3^ and 75% of glioma patients in the United States die within 5 years of diagnosis.^4^ Nonetheless, as the prognosis of gliomas remains heterogeneous, the identification of diagnostic and prognostic markers of glioma is urgently needed to devise more effective individually targeted therapies for this disease.

Cytoplasmic polyadenylation element binding protein 4 (CPEB4) is a sequence-specific RNA-binding protein that participates in translational control. CPEB4 is a member of the CPEB family, which includes CPEB1, CPEB2, CPEB3, and CPEB4, all of which share structure and sequence identity in the C-terminal RNA-binding domain (RBD). CPEB2, CPEB3, and CPEB4 are more closely related to each other than to CPEB1. For example, CPEB2 to 4 exhibit 96% and 25–35% sequence identity in the RBD and N-terminal regulatory regions, respectively, but they share only 45% sequence identity with CPEB1 in the RBD region.^5^ Recent studies suggest that CPEB4 regulates a wide range of biological processes involved in tumor and progression, such as tumor cell growth, vascularization, invasion, and metastasis.^6–8^ Moreover, CPEB4 is overexpressed in some human tumor types, including pancreatic ductal carcinoma and the glioblastoma (WHO grade IV) cell line T98.^6^ However, the CPEB4 expression level in human glioma cell lines and normal brain tissue, the correlation between positive CPEB4 expression and the pathological grade of glioma tissue, and the significance of CPEB4 expression to the clinical prognosis of glioma patients remain unclear. This study aimed to explore the value of the determination of CPEB4 expression for elucidating the mechanism of glioma, guiding clinical diagnosis and treatment, and providing an experimental basis for further investigation. Therefore, we investigated the expression and clinical significance of CPEB4 in glioma tissue of different WHO grades, in normal brain tissue and in human glioma cell lines.

## MATERIALS AND METHODS

### Patients and Tissue Specimens

Paraffin-embedded tissue samples from 229 patients with glioma (WHO I–IV) and 4 freshly prepared samples [B958 (normal brain tissue), B099 (WHO I), B430 (WHO III), and B315 (WHO IV)] were obtained from the archives of the Department of Pathology, Cancer Center, Sun Yat-Sen University, Guangzhou, China, between 1998 and 2008. The tumor specimens were histologically confirmed and selected based on the availability of resected tissue and follow-up data and the lack of preoperative radiation or chemotherapy. Additionally, all samples were ethically approved for use based on informed consent, including 12, 72, 81, and 64 glioma cases classified as WHO I, WHO II, WHO III, and WHO IV (glioblastoma), respectively. Moreover, 41 normal brain tissue specimens, which were resected for the treatment of nonglioma diseases, were examined in this study. Patients whose cause of death was unknown and those who had received neoadjuvant and adjuvant therapy were excluded from our study. Our group of glioma patients included 125 (55%) men and 104 (45%) women, and the clinicopathological characteristics of the tumor sets are described in Tables 1 and 2. The average follow-up duration was 35.59 months (median, 22 months; range, 1–142 months).

**TABLE 1:**
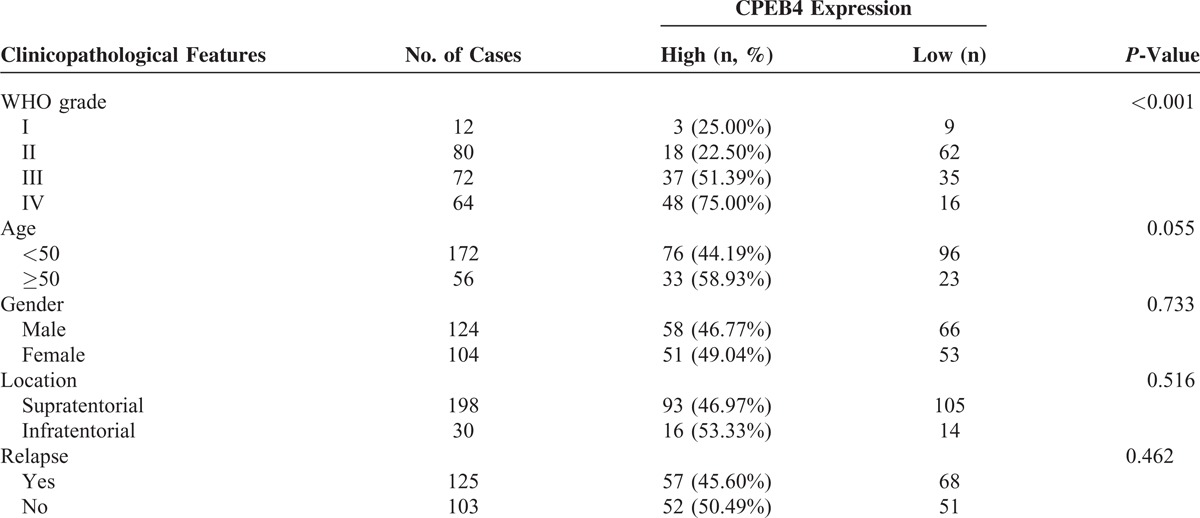
Associations of CPEB4 Expression in Human Glioma Tissues With Different Clinicopathological Features

**TABLE 2:**
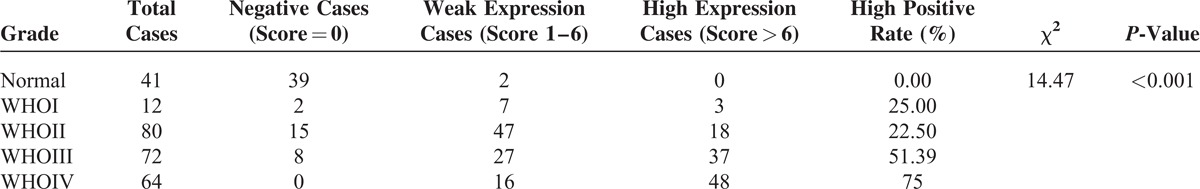
Expression of CPEB4 Protein in 228 Cases With Brain Gliomas and 41 Cases of Normal Brain Tissues

### Tissue Microarray (TMA) Construction

TMAs were constructed as described previously.^9,10^ Briefly, formalin-fixed, paraffin-embedded tissue blocks and corresponding histological H&E-stained slides were overlaid for TMA sampling. A senior pathologist reviewed the slides to determine and label representative areas of viable tumor tissue. Triplicate 1.0-mm diameter cylinders of tissue were punched from selected tumor areas of individual donor tissue blocks and were reembedded in recipient paraffin blocks at defined positions using a tissue arraying instrument (Beecher Instruments, Silver Spring, MD) to control for tumor heterogeneity. The TMA block contained 229 glioma samples, including 12 WHO I, 81 WHO II, 72 WHO III, and 64 WHO IV samples. Subsequently, multiple sections were sliced from the TMA block and mounted on microscope slides. One section from the tissue array block was stained with H&E to confirm that the punches contained tumor tissue.

### Immunohistochemistry (IHC)

Immunohistochemical analysis of CPEB4 expression was performed using a previously described standard technique.^11^ TMA slides were dried overnight at 37°C, dewaxed in xylene, rehydrated using a graded alcohol series, and immersed in 3% hydrogen peroxide for 20 minutes to block endogenous peroxidase activity. The slides were pretreated in antigen retrieval buffer (citrate buffer, pH 6.0, at 100°C for 2 minutes or EDTA buffer, pH 8.0, at 100°C for 2 minutes in a pressure cooker) and then incubated in 10% normal goat serum at room temperature for 10 minutes to reduce nonspecific reactivity. Subsequently, the TMA slides were incubated in a rabbit polyclonal antibody against CPEB4 (1:500; Cell Signaling Technology, Inc., Boston, MA) overnight at 4°C. The slides were rinsed 5 times with 0.01 M phosphate-buffered saline (PBS; pH 7.4) for 10 minutes, and the primary antibodies were detected with a secondary antibody (Envision; Dako, Glostrup, Denmark) for 1 hour at room temperature. Then, the slides were washed in PBS and stained with 3,3-diaminobenzidine (DAB). Finally, the sections were counterstained using Mayer's hematoxylin, dehydrated, and mounted. Nonneoplastic brain tissues were used as controls, and the anti-CPEB4 antibody was replaced with PBS alone as a negative control for immunohistochemical staining.

### Clinicopathological Characteristics and Assessment of IHC

Two pathologists who were blinded to the clinical data independently assessed the expression of the factors assessed via IHC. The scores of the 2 pathologists were compared, and any discrepancies were resolved via the reexamination of the sample by both pathologists to achieve a consensus score. The distribution of factor expression based on IHC was semi-quantitatively assessed by estimating the proportion and the intensity of positively stained tumor cells as demonstrated in previous studies.^11^ Briefly, the adjusted Allred scoring system was applied to evaluate the entire area of every slide via light microscopy. First, a proportion score was assigned using the following 4-point scale: 0, 0% to 5% positive tumor cells; 1, 6% to 25% positive tumor cells; 2, 26% to 50% positive tumor cells; 3, 51% to 75% positive tumors cells; and 4, >75% positive tumor cells. The following 4-point intensity scoring system was used: 0, no staining; 1, weak staining, light yellow; 2, moderate staining, yellowish brown; and 3, strong staining, brown/black. A final immunoreactivity score was obtained for each case by multiplying the proportion and intensity scores. The scores for tumors with multiple cores were averaged. Protein expression was defined as negative (score = 0), weak (score = 1–6), or strong (score>6). We stratified negative or weak CPEB4 protein expression into the low CPEB4 group and strong CPEB4 protein expression into the high CPEB4 group. The association of clinicopathological characteristics such as age, gender and tumor location, grade and recurrence with CPEB4 expression was analyzed.

### Cell Culture

The human glioma cell line SHG-44 (derived from a WHO II glioma), the human glioblastoma cell lines SKMG-4, U87, and T98 and the human lung carcinoma cell line A549 were provided by the State Key Laboratory of Oncology in South China. All cells were cultured at 37°C in 5% CO_2_ in DMEM (Gibco, Carlsbad, CA) supplemented with 10% fetal bovine serum (FBS) (Gibco), 1% penicillin and streptomycin (Sigma, St. Louis, MO).

### Western Blot Analysis

Western blotting was performed in accordance with a previously described protocol.^12^ Cells were collected from flasks and washed 3 times with cold PBS. Tissue samples (3 glioma tissues and 1 normal brain tissue resected for the treatment of nonglioma disease) were ground with liquid nitrogen and lysed at 4°C for 30 minutes in lysis buffer (50 mM Tris, pH 7.4, 100 mM NaCl_2_, 1 mM MgCl_2_, 2.5 mM Na_3_VO_4,_ 1 mM phenylmethylsulfonyl fluoride, 2.5 mM EDTA, 0.5% Triton X-100, 0.5% NP-40, and 5 mg/mL each of aprotinin, pepstatin A, and leupeptin). The lysates were centrifuged at 10,000 *g* for 15 minutes at 4°C. Protein concentrations were determined using a BCA protein assay reagent kit (Pierce, Rockford, Milwaukee, WI, USA) according to the manufacturer's protocol. Forty micrograms of total protein were electrophoresed in a 10% denaturing sodium dodecyl sulfate (SDS) gel and transferred to a polyvinylidene difluoride (PVDF) membrane. The PVDF membrane was incubated in blocking buffer (PBS containing 5% nonfat milk) for 2 hours at room temperature, followed by incubation in a rabbit polyclonal antibody against CPEB4 (Cell Signaling Technology, Inc.) diluted 1:500 overnight with gentle shaking. The membrane was washed twice with PBS for 5 minutes and incubated in the secondary antibody horseradish peroxidase-conjugated goat antirabbit/antimouse immunoglobulin G (Santa Cruz Biotechnology, Dallas Texas, USA) diluted 1:2000 for 2 hours at room temperature. GAPDH was detected using a rabbit polyclonal antibody (Santa Cruz Biotechnology) as a loading control. The experiments were repeated 3 times.

### Total RNA Isolation and Real-Time PCR

Total RNA was extracted from cells and tissues using Trizol reagent (Invitrogen, Thermo Fisher Scientific Inc. Waltham, MA, USA) according to the manufacturer's instructions. The RNA was pretreated with DNase, and single-stranded cDNA was synthesized using the SuperScript First Strand Synthesis System (Life Technologies, Thermo Fisher Scientific Inc. Waltham, MA, USA) according to the manufacturer's instructions. CPEB4 was used for real-time PCR to amplify the cDNA from 4 glioma cell lines, 1 lung carcinoma cell line and 4 patient tissues. All real-time quantitative RT-PCR reactions were performed using a SYBR Green master mix kit and an ABI PRISM 7500 sequence detection system. GAPDH was used as an internal loading control for quantitative RT-PCR. The nucleotide sequences of the forward and reverse primers for the CPEB4 gene^6^ and the GAPDH gene are listed in Table 3.

**TABLE 3:**
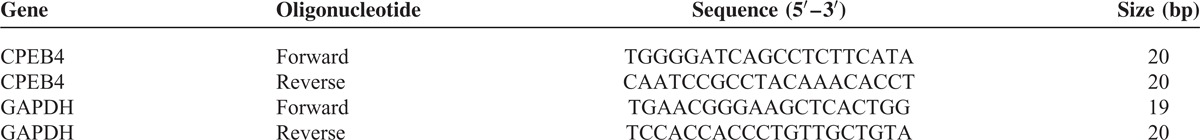
Sequences of Primers Used in Polymerase Chain Reaction

### Statistical Analysis

SPSS version 16.0 was applied for statistical evaluations. The relationship between CEBP4 protein expression and the clinicopathological characteristics of the glioma patients was estimated using the χ^2^-test, and Spearman's rank correlation analysis was used to analyze the correlation between the level of CPEB4 expression and the WHO grade. Overall survival (OS) was assessed using the Kaplan–Meier method, and the log-rank test was used to analyze the resulting survival curves. Multivariate Cox regression analysis was performed to identify the independent factors that significantly impacted patient survival. A probability value less than 0.05 (*P* < 0.05) was considered to be significant.

## RESULTS

### Relatively High Levels of CPEB4 Protein and mRNA Expression in Glioma Cell Lines and Tissues

**FIGURE 1:**
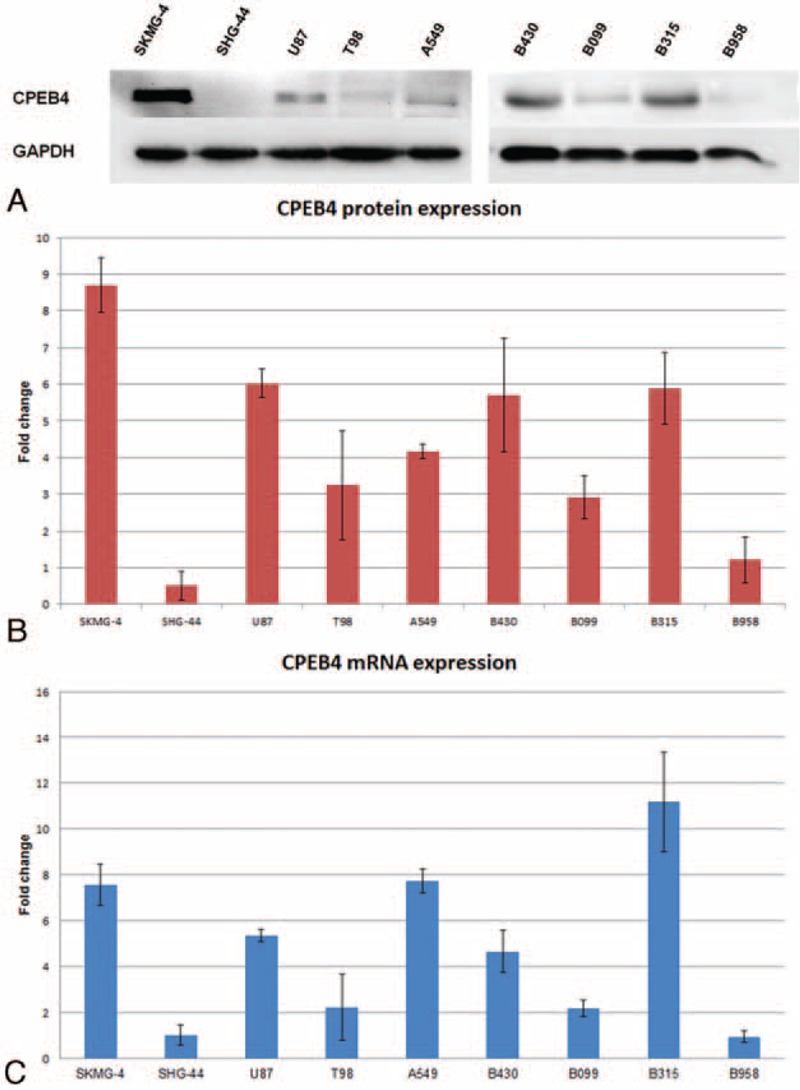
The expression of CPEB4 in glioma samples based on real-time PCR and Western blotting. From left to right: the human glioblastoma cell line SKMG-4, the human glioma cell line SHG-44, the human glioblastoma cell line U87, the human glioblastoma cell line T98, the human lung carcinoma cell lines A549 (positive control), freshly prepared sample B430 (WHO III), freshly prepared sample B099 (WHO I), freshly prepared sample B315 (WHO IV), and freshly prepared sample B958 (normal brain tissue). (A) Expression of the CPEB4 protein in glioma cell lines and freshly prepared tissue samples. (B) Fold change in CPEB4 protein expression in glioma cell lines and freshly prepared tissue samples. (C) Fold change in CPEB4 mRNA expression in glioma cell lines and freshly prepared tissue samples.

Western blotting and real-time PCR analyses revealed a clearly higher level of CPEB4 expression in 3 glioblastoma cell lines (SKMG-4, U87, and T98) than in a glioma cell line (SHG44). The human lung carcinoma cell line A549 was set as a positive control for comparison (Figure 1). Our results clearly demonstrated a relatively higher expression level of CPEB4 in freshly prepared high-grade glioma tissue samples B430 (WHO III) and B315 (WHO IV) than in the low-grade glioma sample B099 (WHO I). The level of CPEB4 expression was extremely weak in the adjacent nonneoplastic brain tissue B958 (Figure 1).

### Overexpression of CPEB4 Proteins in Human Glioma Tissues Based on IHC

**FIGURE 2:**
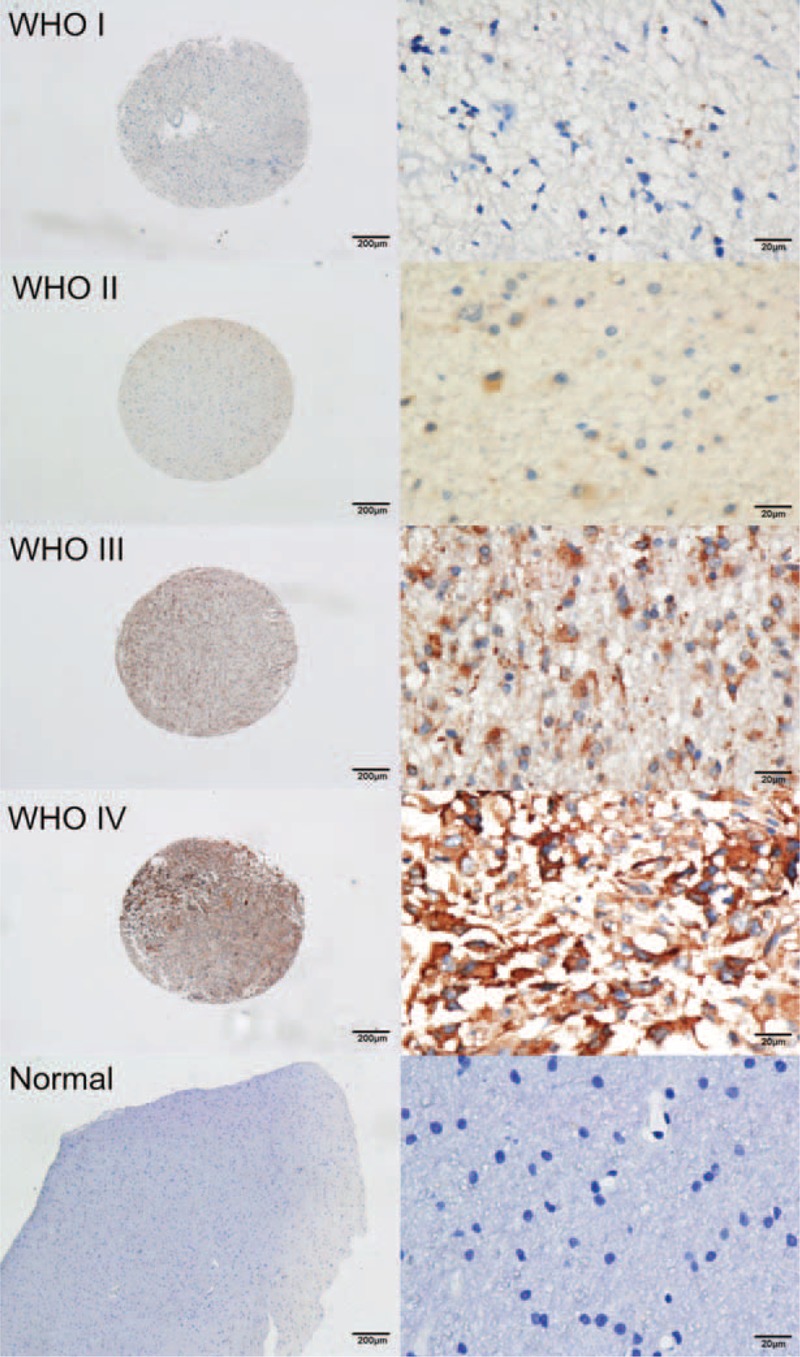
Representative sections of CPEB4 immunoreactivity in glioma and nonneoplastic brain tissues (left, ×20; right, ×400). The CPEB4 protein was primarily expressed in the cytoplasm, as demonstrated by brownish yellow staining.

**FIGURE 3:**
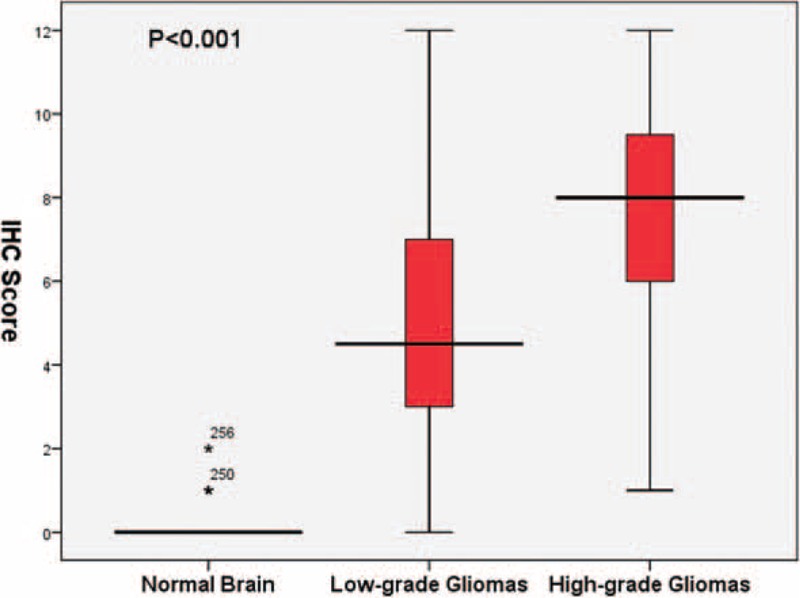
The box plots demonstrate the range of CPEB4 expression (based on the immunohistochemical score) for each group (normal brain tissues, N = 41; low-grade glioma tissues, N = 92; and high-grade glioma tissues, N = 136).

The positive expression of CPEB4 was examined in 203/228 (89.04%) of gliomas in the examined cohort (1 case of WHO II was dropped from the TMA analysis), and we confirmed no or extremely weak CPEB4 expression in 2/41 (4.88%) normal brain tissue samples (Figure 2 and Table 2). Notably, positive CPEB4 expression was observed in 64/64 (100%) glioblastoma (WHO IV) samples (Table 2). The expression of CPEB4 in high-grade glioma was higher than that in low-grade glioma, and highly positive CPEB4 expression in the glioma tissue was significantly associated with a more aggressive tumor phenotype. Spearman's rank correlation analysis revealed a positive correlation between high CPEB4 expression and the WHO grade (r = 0.774, *P* < 0.01) (Figure 3 and Table 2).

### Association of CPEB4 Expression With the Clinicopathological Characteristics and the Survival of Glioma Patients

**FIGURE 4:**
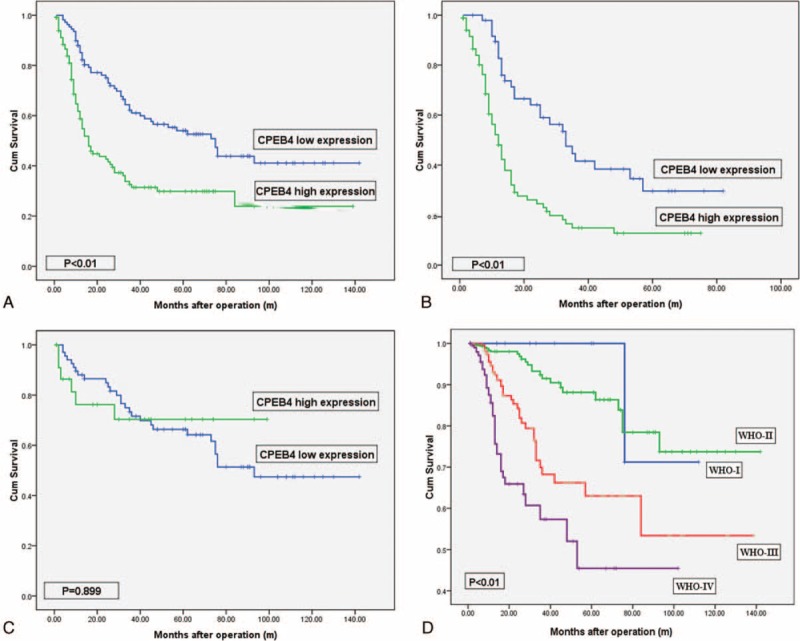
Kaplan–Meier survival curves of glioma patients. (A) The OS of all glioma patients with CPEB4 protein expression: high versus low. (B) The OS of high CPEB4 protein expression and low CPEB4 protein expression in high-grade glioma patients. (C) The OS of high CPEB4 protein expression and low CPEB4 protein expression in low-grade glioma patients. (D) The OS of glioma patients with high WHO grade (III–IV) was significantly lower than that of glioma patients with low WHO grade (I–II).

Table 1 summarizes the associations between CPEB4 protein overexpression and the clinicopathological characteristics of human glioma cases. The upregulation of CPEB4 protein was significantly associated with advanced WHO grade (*P* < 0.01, Table 1). No significant association of CPEB4 with age, gender or tumor location or recurrence was observed (all *P* > 0.05, Table 1). Kaplan–Meier plots showed that the total glioma patients with high CPEB4 expression exhibited significantly shorter OS (*P* < 0.01) than those with low CPEB4 expression (Figure 4A). More importantly, high CPEB4 expression indicated a poorer survival in high-grade glioma patients (*P* < 0.01) (Figure 4B) while it showed no statistical significance in low-grade glioma patients (*P* = 0.899) (Figure 4C). Moreover, we can confirm the correctness of the data for the fact that the OS of glioma patients with an advanced WHO grade (III–IV) was significantly lower than that of those with a low WHO grade (I–II) (Figure 4D). The patients with high or low CPEB4 expression exhibited a median OS duration of 21 and 76 months, respectively (*P* < 0.01, log-rank test, Table 4). Multivariate Cox regression analyses indicated that CPEB4 expression was an independent prognostic factor of poor survival in glioma patients. Other clinical parameters examined in the stepwise Cox proportional hazards model are presented in Table 5.

**TABLE 4:**
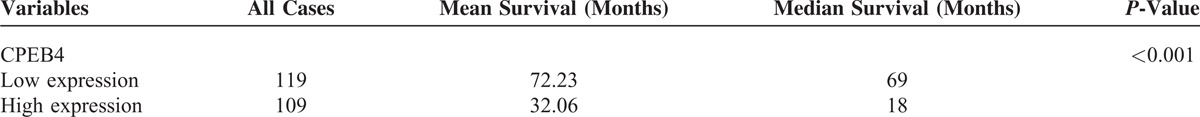
Univariate Analysis of CPEB4 Expression in Glioma Patients (Log-Rank Test)

**TABLE 5:**
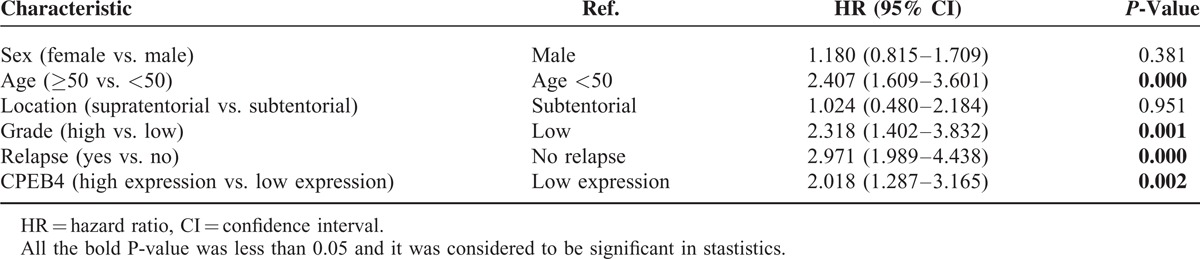
COX Multivariate Analysis of Clinicopathological Parameters and Overall Survival

## DISCUSSION

CPEB is a highly conserved RNA-binding protein that promotes the elongation of the polyadenine tail of mRNA.^13^ CPEB participates in a series of important processes, including stem cell development, cell differentiation, cell senescence, and synaptic plasticity. CPEB4, which is located at chromosome 5q35.2, is a member of the CPEB family. The CPEB4 protein consists of 729 amino acids and exhibits a molecular weight of 80.2 kDa, and CPEB4 plays a key role in gene transcriptional regulation during tumor development. Ortiz-Zapater et al^6^ found that CPEB4 was overexpressed in pancreatic ductal adenocarcinomas, supporting tumor growth, vascularization, and invasion. CPEB4 is associated with a large number of CPE-containing mRNAs that are potential targets for tumor-specific translational regulation. Additionally, these investigators used CPEB4 shRNA to silence its expression and demonstrated that CPEB4 promotes tumor growth and vascularization in the T98 cell line and in the RWP-1 pancreatic ductal adenocarcinoma cell line.

The present study initially established that CPEB4 was expressed at a higher level in a panel of glioma cell lines and tissues than in a positive control cell line or in adjacent brain tissues. We next performed IHC to examine the dynamics of CPEB4 expression in glioma tissues of different stage based on complete follow-up data and in normal brain tissues. Our results demonstrated that the mean staining intensity of CPEB4 in glioma tissue, especially high-grade glioma tissue, was significantly greater than that in normal brain tissue. We found that CPEB4 was barely expressed in normal brain astrocytes (2/41, 4.9%). The positive expression rate of CPEB4 in the glioma cells was increased (203/228, 89.04%), and the positive expression rate of CPEB4 in glioblastoma cells was extremely high (64/64, 100%). CPEB4 upregulation was significantly associated with higher WHO grade. These observations strongly suggest that the evaluation of CPEB4 expression using IHC may enable the discrimination between glioma and nonneoplastic brain tissue. More importantly, our data showed that CPEB4 overexpression alone appeared to be an independent prognostic factor for OS in glioma patients, which suggests that the detection of CPEB4 may be valuable for the design of optimal individualized treatments and for the identification of patients who may or may not benefit from close monitoring after surgery. Nevertheless, the identification of the exact signaling pathway and the best use of CPEB4 as a marker to stratify cancer patients for personalized treatment remain critical goals. Additional research is required to confirm our findings. Recently, Peng et al^14^ demonstrated that dendritic cells transduced with a CPEB4 expression vector produced specific cytotoxic lymphocytes that lysed the syngeneic murine glioma cell line GL261 and that displayed increased IFN-γ secretion. These CPEB4-specific cytotoxic lymphocytes exerted no detectable lytic effect on autologous lymphocytes in vitro. These studies demonstrated that dendritic cells transduced with a CPEB4 expression vector exhibited enhanced antitumor function and induced antitumor immune responses in vitro and in vivo, providing great promise for the treatment of cancer, especially glioma, in the future. Zaccara et al^15^ confirmed that the CPEB4 gene was directed by p53 transcriptional targets and that p53 impacted its own translation fitness and functions via CPEB4. However, the impact of CPEB4 on p53 is unclear and hypothetical because it is based on results concerning the related CPEB1 protein.^16^ Chang and Huang^17^ found that CPEB4 interacted with the adaptor protein vinexin, affecting cell migration. Arsenite-activated JNK signaling enhanced the CPEB4–vinexin interaction to promote stress granule assembly and cell survival under stress. Additionally, Hu et al^18^ found that CPEB4 was strongly induced by the important erythroid-related transcription factors Gata1 and Tal1 and that 1 CPEB4 target gene is CDK6, an important protein in cell cycle regulation and cancer development. These investigators proposed that the control of CPEB4 gene expression during mammalian cell differentiation was essential for cell proliferation and apoptosis. However, the exact CPEB4 signaling pathway that is involved in cancer progression remains unknown.

In conclusion, our results strongly suggest that CPEB4 overexpression promotes glioma progression and that CPEB4 may serve as a biomarker of a more aggressive glioma phenotype. Our data identify the potential clinical value of CPEB4 expression assessment, which will aid in the prediction of clinical outcomes of glioma patients.

## References

[1] Goodenberger ML Jenkins RB Genetics of adult glioma. *Cancer Genet* 2012; 205:613–621. 23238284 10.1016/j.cancergen.2012.10.009

[2] Dunbar E Yachnis AT Glioma diagnosis: immunohistochemistry and beyond. *Adv Anat Pathol* 2010; 17:187–201. 20418673 10.1097/PAP.0b013e3181d98cd9

[3] Liu Q Li G Li R IL-6 promotion of glioblastoma cell invasion and angiogenesis in U251 and T98G cell lines. *J Neurooncol* 2010; 100:165–176. 20361349 10.1007/s11060-010-0158-0

[4] Starkweather AR Sherwood P Lyon DE A biobehavioral perspective on depressive symptoms in patients with cerebral astrocytoma. *J Neurosci Nurs* 2011; 43:17–28. 21338041 10.1097/jnn.0b013e3182029859 PMC3732744

[5] Huang YS Kan MC Lin CL CPEB3 and CPEB4 in neurons: analysis of RNA-binding specificity and translational control of AMPA receptor GluR2 mRNA. *EMBO J* 2006; 25:4865–4876. 17024188 10.1038/sj.emboj.7601322 PMC1618119

[6] Ortiz-Zapater E Pineda D Martinez-Bosch N Key contribution of CPEB4-mediated translational control to cancer progression. *Nat Med* 2012; 18:83–90. 22138752 10.1038/nm.2540

[7] Xu H Liu B CPEB4 is a candidate biomarker for defining metastatic cancers and directing personalized therapies. *Med Hypotheses* 2013; 81:875–877. 24045092 10.1016/j.mehy.2013.08.030

[8] D’Ambrogio A Nagaoka K Richter JD Translational control of cell growth and malignancy by the CPEBs. *Nat Rev Cancer* 2013; 13:283–290. 23446545 10.1038/nrc3485

[9] Kononen J Bubendorf L Kallioniemi A Tissue microarrays for high-throughput molecular profiling of tumor specimens. *Nat Med* 1998; 4:844–847. 9662379 10.1038/nm0798-844

[10] Cai MY Zhang B He WP Decreased expression of PinX1 protein is correlated with tumor development and is a new independent poor prognostic factor in ovarian carcinoma. *Cancer Sci* 2010; 101:1543–1549. 20367640 10.1111/j.1349-7006.2010.01560.x PMC11159430

[11] Wang JP Hu WM Wang KS Upregulation of C-X-C chemokine receptor type 1 expression is associated with late-stage gastric adenocarcinoma. *Exp Ther Med* 2012; 4:55–60. 23060922 10.3892/etm.2012.568 PMC3460258

[12] Hu W Wang J Luo G Proteomics-based analysis of differentially expressed proteins in the CXCR1-knockdown gastric carcinoma MKN45 cell line and its parental cell. *Acta Biochim Biophys Sin (Shanghai)* 2013; 45:857–866. 23924695 10.1093/abbs/gmt086

[13] Hake LE Richter JD CPEB is a specificity factor that mediates cytoplasmic polyadenylation during Xenopus oocyte maturation. *Cell* 1994; 79:617–627. 7954828 10.1016/0092-8674(94)90547-9

[14] Peng W Nan Z Liu Y Dendritic cells transduced with CPEB4 induced antitumor immune response. *Exp Mol Pathol* 2014; 97:273–278. 24927871 10.1016/j.yexmp.2014.06.001

[15] 2014; Zaccara S Tebaldi T Pederiva C p53-directed translational control can shape and expand the universe of p53 target genes. 21:1522–1534. 10.1038/cdd.2014.79 PMC4158691 24926617

[16] Fernandez-Miranda G Mendez R The CPEB-family of proteins, translational control in senescence and cancer. *Ageing Res Rev* 2012; 11:460–472. 22542725 10.1016/j.arr.2012.03.004

[17] Chang YW Huang YS Arsenite-activated JNK signaling enhances CPEB4-Vinexin interaction to facilitate stress granule assembly and cell survival. *PLoS One* 2014; 9:e107961. 25237887 10.1371/journal.pone.0107961 PMC4169592

[18] Hu W Yuan B Lodish HF Cpeb4-mediated translational regulatory circuitry controls terminal erythroid differentiation. *Dev Cell* 2014; 30:660–672. 25220394 10.1016/j.devcel.2014.07.008 PMC4182162

